# Molgenis-impute: imputation pipeline in a box

**DOI:** 10.1186/s13104-015-1309-3

**Published:** 2015-08-19

**Authors:** Alexandros Kanterakis, Patrick Deelen, Freerk van Dijk, Heorhiy Byelas, Martijn Dijkstra, Morris A Swertz

**Affiliations:** Department of Genetics, Genomics Coordination Center, University Medical Center Groningen and University of Groningen, Genetics, UMCG, PO Box 30 001, 9700 RB Groningen, The Netherlands

**Keywords:** Imputation, Genotyping, GWAS

## Abstract

**Background:**

Genotype imputation is an important procedure in current genomic analysis such as genome-wide association studies, meta-analyses and fine mapping. Although high quality tools are available that perform the steps of this process, considerable effort and expertise is required to set up and run a best practice imputation pipeline, particularly for larger genotype datasets, where imputation has to scale out in parallel on computer clusters.

**Results:**

Here we present MOLGENIS-impute, an ‘imputation in a box’ solution that seamlessly and transparently automates the set up and running of all the steps of the imputation process. These steps include genome build liftover (liftovering), genotype phasing with SHAPEIT2, quality control, sample and chromosomal chunking/merging, and imputation with IMPUTE2. MOLGENIS-impute builds on MOLGENIS-compute, a simple pipeline management platform for submission and monitoring of bioinformatics tasks in High Performance Computing (HPC) environments like local/cloud servers, clusters and grids. All the required tools, data and scripts are downloaded and installed in a single step. Researchers with diverse backgrounds and expertise have tested MOLGENIS-impute on different locations and imputed over 30,000 samples so far using the 1,000 Genomes Project and new Genome of the Netherlands data as the imputation reference. The tests have been performed on PBS/SGE clusters, cloud VMs and in a grid HPC environment.

**Conclusions:**

MOLGENIS-impute gives priority to the ease of setting up, configuring and running an imputation. It has minimal dependencies and wraps the pipeline in a simple command line interface, without sacrificing flexibility to adapt or limiting the options of underlying imputation tools. It does not require knowledge of a workflow system or programming, and is targeted at researchers who just want to apply best practices in imputation via simple commands. It is built on the MOLGENIS compute workflow framework to enable customization with additional computational steps or it can be included in other bioinformatics pipelines. It is available as open source from: https://github.com/molgenis/molgenis-imputation.

**Electronic supplementary material:**

The online version of this article (doi:10.1186/s13104-015-1309-3) contains supplementary material, which is available to authorized users.

## Background

Genotype imputation uses densely typed reference haplotypes to infer untyped genotypes [1]. The resulting imputed datasets are commonly used in meta-analyses to gain statistical power or for the fine-mapping of association signals [2]. Modern imputation methods enable inference of many types of genetic variation, including single nucleotide polymorphisms (SNPs), insertions and deletions [3].

Imputation has been widely adopted as it has led to the identification of additional associations [4] and has allowed combination studies from different genotyping platforms [5, 6] contributing to a meta-analysis [7]. Another benefit is the fine mapping of association signals: since detected regions are not usually located in a functional region but due to linkage disequilibrium (LD) they are highly associated with the true causal variant that might not have been assayed at all. Finally, it has been suggested [8] that by using imputation we can enrich rare variants with large effect that contribute significantly to the ‘missing heritability’ of complex traits such as lipid profiles.

Today, there are high quality tools that perform imputation, including Minimac [9], BEAGLE [5] and IMPUTE2 [2]. Many studies have evaluated their performance regarding parameters such as the genotype platform of the imputed study, the number of SNPs, ethnicity of the samples, reference panels, LD structure, allele frequency of variants, improvement of statistical power in genome-wide association studies (GWAS), and enhancement in the identification of causal variants [10–14].

The general consensus from these comparisons is that these three imputation tools exhibit a similar performance in terms of the estimated correlation between the imputed and true genotypes. Although all the tools could be easily added, we decided to include IMPUTE2 because of previous experience in our team, reported marginal benefits in terms of unrelated reference panels [14], and execution times when using prephased data [15]. The phasing software that we selected was SHAPEIT [16], mainly because it integrates nicely with IMPUTE2 and is highly recommended by the authors of IMPUTE2 [9]. The authors of SHAPEIT also demonstrate an improved phasing accuracy compared to other methods.

Regardless of the choice of imputation tool, considerable work has to be done before any of these can be used in a full pipeline. The reason for this is that these tools require pre- and post-processing steps such as liftovering, quality control, and splitting/merging of data in order to work effectively. Liftover is the process of changing the genomic positions of a dataset from one version of a genome assembly to another. It is very common that a study panel is aligned to a different build of a genome assembly than the reference panel. This pre-processing step is essential and precedes all genomic studies that include a study combining more than one dataset.

Existing bioinformatics pipeline management systems have a limited coverage for genotype imputation. For example, Galaxy [17] does not include imputation methods in its public database. Other workflow management systems like Taverna [18] and Ergatis [19] offer a thorough and complete toolset for describing bioinformatics workflows, but lack specific cases for genotype imputation. To our knowledge, the only bioinformatics solution relevant to imputation is GRIMP [20], but rather than performing imputation, it focuses on the analysis of the GWAS data that usually follows it. Moreover, setting up such advanced workflow systems requires more time and skill than most genetics researchers have available.

Here we present MOLGENIS-impute, a simple command line tool to run complete genotype imputation pipelines on local servers and a variety of HPC environments. This tool is for geneticists and lab bioinformaticians who simply want to perform an imputation with minimal overhead of discovering, installing and configuring tools, while ensuring best practices are followed and applying proper quality control. The steps covered are aligning markers to the same genomic reference as the reference panel (by default hg19), applying quality control to check for genomic strand inconsistencies between the study and the reference panel, phasing the study panel, splitting the study panel into multiple chromosomal and sample chunks, and merging the resulted imputed dataset. The pipeline can be executed either on a local or cloud server for smaller studies, or on an HPC cluster or grid environment for larger efforts. The solution we offer employs a fail-safe approach regarding any failures that might occur during execution. The user interacts with a simple python script via command line options. Although the required tools and commands that are necessary to execute this script are relatively common in a Linux installation, we have included a list of these tools and installation instructions in Additional file 1.

## Methods

Before imputation, researchers normally spend a considerable amount of time in setting up a pipeline that includes the necessary pre- and post-processing steps, such as liftovering, quality control, and splitting/merging of data. The first main component of our approach is a single script that downloads, configures and installs all the necessary tools, data and scripts (Table 1). After careful evaluation, we selected the IMPUTE2 and SHAPEIT2 tool family for this implementation, but our pipelines can be extended to include other tools as well. The second main component is the automation of all the necessary steps. Here we use the MOLGENIS-compute package to auto-generate simple shell scripts ready for execution on a local server or for submission to a cluster or grid [21]. The only prerequisite is that the study panel should be in the default standard PLINK PED/MAP format or the binary equivalent BED/BIM/FAM [22]. Our pipeline consists of the following steps:

**Table 1:** Tools and data installed during MOLGENIS-impute set up

Step	Tool	Version	Usage
Set up	Molgenis-compute	0.0.1	Manage scripts, handle parameters, submit to HPC
	Molgenis-pipelines	0.1.0	Imputation BASH scripts and Pipeline in CSV format
Step 1	Liftover	20120905	Change genomic reference of study panel to the one used by the reference panel (by default from hg18 to hg19)
	PLINK	1.07	Update marker position of input files during liftover step
Step 2	SHAPEIT	v2.r644	Phasing of study panel
Step 3	Genotype Harmonizer	1.3.1	Perform quality control
Step 4	Bash script		Split data in sample chunks
Step 5	Impute2	v2.3.0	Main imputation tool
Prepare reference panel	vcftools	0.1.11	Convert reference panel from VCF format to IMPUTE2
	tabix	0.2.6	Compress reference panel VCF files and build index

Step	Data	Version	Usage
Step 1	Hg18 to hg19 chain file		Map positions between hg18 and hg19 genomic reference
Step 2, 3	Subset (chromosome 1, first 10 Mbp) of HapMap data	v.3 release 2	Example study panel for imputation
Step 3	Subset (chromosome 1, first 10 Mbp) of 1,000 Genomes project	GIANT release	Example reference panel for imputation
Step 3	Recombination map for hg19		Calibration of hidden Markov model [13]

- *Step 1* is the liftovering, or the conversion of the positions of the study panel to those used by the reference panel. By default, this optional step converts from UCSC hg18 to UCSC hg19 genomic assembly. Alternatively, a user can specify a chain file in order to perform liftover between other builds of the genome. Chain files contain a mapping of the positions between two different genome assemblies. We provide a list of chain file repositories on the documentation page for MOLGENIS-impute.
- *Step 2* is the phasing using SHAPEIT2, with which we infer the haplotype structure of the genotype data. Although this step is not necessary for imputation per se, it increases the imputation quality and it significantly speeds up the process. Especially when multiple imputation tasks have to be performed, phasing of the study panel only needs to be done once. This is useful when a new version of a reference panel becomes available.
- *Step 3* is the quality control step that guarantees that data in the study panel are aligned to the same strand as the reference panel. Alignment of G/C and A/T variants is performed by assessing the LD structure using Genotype Harmonizer [23]. This tool removes SNPs from the study when strand correction cannot be applied (for example, an A/T SNP in the study that exists as A/C in the reference panel). It also generates a log file of all the performed checks that includes all the removed markers.
- *Step 4* is the splitting of the study panel into sample and chromosomal chunks. By default, each chunk contains no more than 5 Mbp length of markers, as recommended by the IMPUTE2 software. The number of samples that each chunk has is a value between 500 and 1,000, and is devised during execution so that chunks have an approximately equal number of samples. This two-dimensional splitting is an essential step in order to handle the enormous computation that is usually required and to scale the imputation process effectively in an HPC environment.
- *Step 5* is the actual imputation. IMPUTE2 employs an agnostic approach regarding the population composition of the reference panel and offers the ability to combine multiple reference panels. Upon completion of all imputation steps, we concatenate the resulting sample and chromosomal chunks for downstream analysis. IMPUTE2 generates two main results files. The first contains the posterior genotype probabilities and the second contains quality metrics per imputed marker. Merging sample chunks for the posterior genotype probabilities is trivial, since sample splitting does not affect them. Unfortunately this does not hold for the quality metrics. To overcome this, we re-compute IMPUTE2’s quality metrics for the concatenated sample files. These quality metrics and the respective formulas are presented in Additional file 2.

### Sample study and reference panel

The installation also contains a sample study and reference panel. The study panel is a subset of 100 samples from the HapMap project (version 2, release 3) and it contains all markers from 1 to 10 million bp in chromosome 1. This dataset contains 4,836 markers. The sample reference panel contains all 1,092 samples of the GIANT release of the 1,000 Genomes Project reference panel. This dataset contains only the markers from 1 to 15 million bp in chromosome 1, namely 88,650 markers.

## Implementation

All computational steps are defined as templates of BASH scripts for the MOLGENIS compute system. BASH is the default shell environment in many modern Linux distributions, and BASH scripts are lightweight and easily embedded into external tools. The designed order of the steps in the pipeline and the input/output parameters of each step are defined in a set of CSV files [21]. MOLGENIS compute has specific mechanisms to accommodate the scripts to different backends (e.g. local, PBS, grid). Based on all of the above, MOLGENIS compute generates the actual analysis scripts. The input and output variables of these scripts are passed as BASH variables. In the header of each generated script there are definitions for the system requirements of this particular step to the specific computer cluster/grid environment. These parameters are the desired number of CPUs, amount of memory, and execution time. Additional documentation is available on https://rawgit.com/molgenis/molgenis-compute/master/molgenis-compute-core/README.html.

The imputation scripts belong to the MOLGENIS-pipelines collection, which also includes scripts and pipelines for next-generation sequencing (NGS) and GWAS analysis. They are hosted in a separate git repository: https://github.com/molgenis/molgenis-pipelines. For imputation, interested readers can check the pipeline at the following directory of the git repository: ‘compute5/Imputation_impute2’.

MOLGENIS-compute offers a mechanism to parse these template files and generate executable scripts. These scripts are adjusted for the execution environment that is specified in the –backend command line parameter. So far, the available options are ‘local’, ‘pbs’ or ‘grid’ and are explained in the ‘usage’ section. Since the distribution and availability of computation resources varies among different HPC environments, it tries to maximize the utilization of resources. Moreover, MOLGENIS-compute handles iterations in the pipeline (for example, for each chromosome), orders the scripts in the correct order and generates a submission script, named submit.sh that, when executed, submits the complete pipeline to the user-defined HPC environment [21]. All these orchestration actions take place without needing any user interaction.

**Fig. 1:**
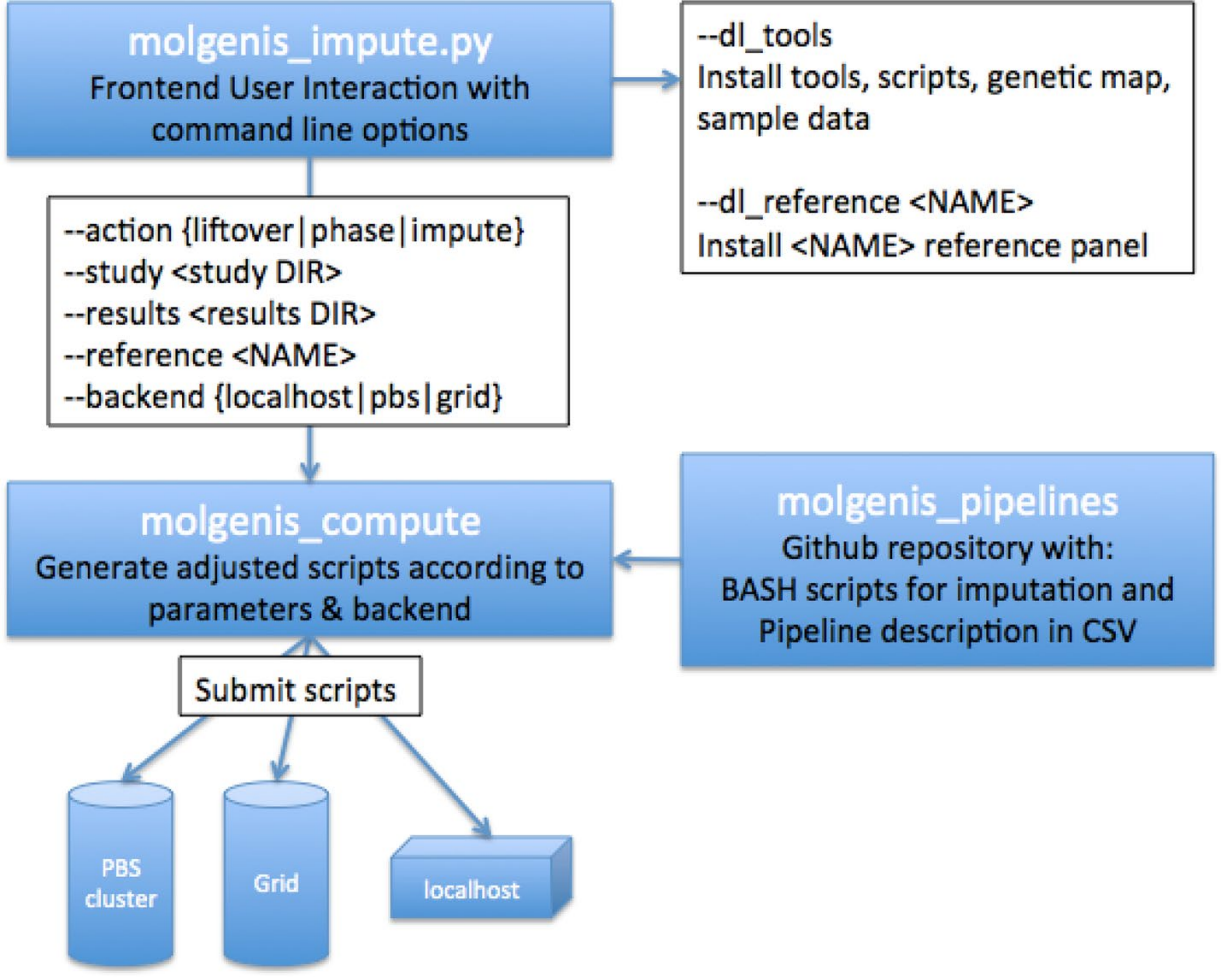
Outline of MOLGENIS-impute architecture. $\tt {molgenis\_impute.py}$ is the python script with which the user interacts. The script can either install tools and reference panels or use MOLGENIS-compute to create and submit imputation scripts. The imputation BASH scripts and description of the pipeline are in a separate git repository.

Finally, we have wrapped the user interaction with the pipeline within a single python script with simple command line arguments. However, all the generated scripts as well as the tool output are still accessible for inspection and review, if needed. To ease installation and usage we have wrapped all the essential operations in a molgenis-impute.py python script that automatically installs all the necessary components on ‘setup’ (see Table 1) and also eases running of the pipeline (see usage, below). Figure 1 depicts the main architecture and functionality of this python script.

## Results and discussion

We first describe the usage of MOLGENIS-impute and then discuss the practical issues, including installation on Amazon EC2 cloud.

### Usage

To install MOLGENIS-impute, clone the following git repository:

- $${\tt{git\,\,clone\,\,git@github.com:molgenis/molgenis-imputation.git}}$$

To download and set up all necessary tools, genetic map and example data (Table 1) run:

- $$\tt {python\,\,molgenis-impute.py\,\,--dl\_{tools}}$$

The tools and scripts that this command installs are compatible with any modern 64 bit Linux operating system. For a complete list of system and software dependencies, see Additional file 1.

The command to list all reference panels that are either installed or available for download is:

- $$\tt {python\,\,molgenis-impute.py\,\,--list}$$

So far, the following reference panels are available for download:

- GIANT.phase1_release_v3.20101123: This is a reference panel prepared from the GIANT consortium [24]. It contains all 1,092 samples from the 1,000 Genomes Project, excluding monomorphic and singleton sites.
- GIANT.metabo.phase1_release_v3.20101123: This is a Metobochip- [25] specific reference panel that focuses on well-imputed, fine-mapped regions.
- 1,000_Genomes_phase3_build37: This dataset is based on sequence data from 2,504 samples from the 1,000 Genomes Project, phase 3 [26].

The command to download a reference panel is:

- $${\tt{python\,\,molgenis-impute.py\,\,--dl\_{reference} <NAME>}}$$

**Fig. 2:**
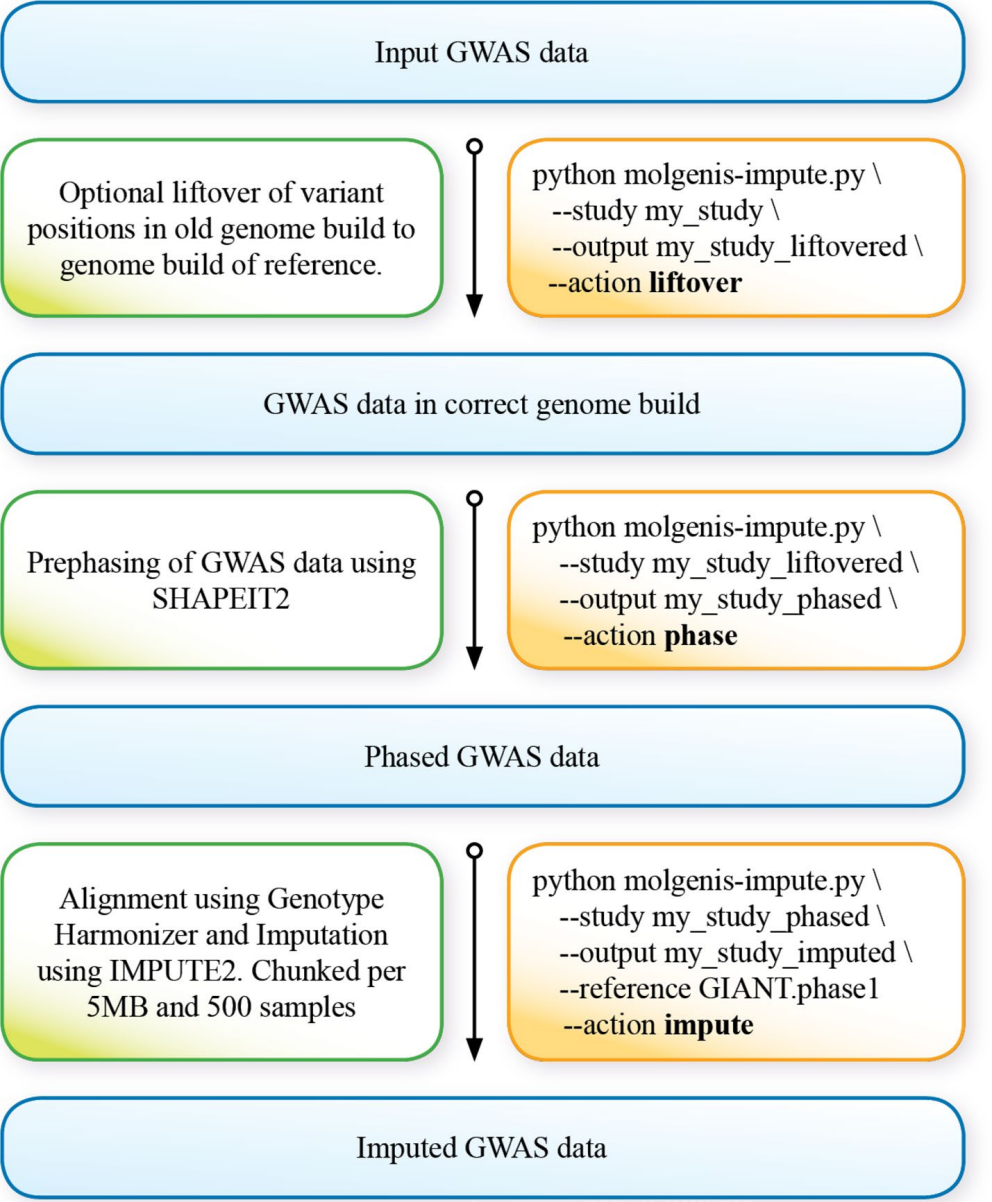
MOLGENIS-impute’s workflow of three steps. Liftovering, phasing and imputation. *Rectangles* on the *left* contain a description of each step and on the *right* a respective demo python command.

We also include commands to install and use an arbitrary imputation reference panel. After these steps, a user can continue with the following steps, depicted in Fig. 2.

Liftovering (optional step):

- $$\tt {python\,\,molgenis-impute.py\,\, \backslash}$$
- $$\tt {\,\,\,\,\,\,\,\,--action\,\,liftover\,\, \backslash}$$
- $$\tt {\,\,\,\,\,\,\,\,--study <input\,\,directory\,\,with\,\,PED/MAP\,\,files> \backslash}$$
- $$\tt {\,\,\,\,\,\,\,\,--output <liftover\,\,results\,\,directory\,\,(PED/MAP)\,\, files>}$$

Phasing:

- $$\tt {python\,\,molgenis-impute.py\,\, \backslash}$$
- $$\tt {\,\,\,\,\,\,\,\,--action\,\,phase \,\,\backslash}$$
- $$\tt {\,\,\,\,\,\,\,\,--study <liftover\,\,results\,\,directory\,\,(PED/MAP)\,\,files> \backslash}$$
- $$\tt {\,\,\,\,\,\,\,\,--output <phasing\,\,results\,\,directory>}$$

Imputing:

- $$\tt {python\,\,molgenis-impute.py\,\, \backslash}$$
- $$\tt {\,\,\,\,\,\,\,\,--action\,\,impute\,\, \backslash}$$
- $$\tt {\,\,\,\,\,\,\,\,--study <phasing\,\,results\,\,directory> \backslash}$$
- $$\tt {\,\,\,\,\,\,\,\,--output <imputation\,\,results\,\,directory>}$$

Many additional options exist for refining the presented steps covering all possible options of IMPUTE2 and SHAPEIT2. The options $\tt {-action\,\,liftover\_phase\_impute}$ and $\tt {liftover\_phase\_impute}$ can be used in order to combine the presented steps in a single run. Moreover, liftovering and phasing commands also accept binary PLINK files (BED/BIM/FAM). Detailed documentation is available on the tool’s site. Execution can take place on three different environments, according to the $\tt {--backend}$ parameter:

*local* This is the default option. The scripts are adjusted for a single CPU, 64 bit local computer with a Linux operating system. This option does not generate any special HPC headers and is intended mainly for testing purposes.

*pbs* Adds headers that allow submission to, for example, Portable Batch System (PBS) [27] or Sun Grid Engine (SGE) clusters.

*grid* Adds headers that allow the submission to a grid middleware, such as glite-WMS grid scheduler. Resources are managed with Storage Resource Management (SRM) [28] and data transfer and submission is managed with Job Description Language (JDL). In order to achieve execution in all nodes of the grid, we employ the MOLGENIS pilot job solution [29], where workflow deployment (i.e. tool availability) is achieved by reusing the environment modules package [30]. Data transfer and pipeline monitoring are hidden in pilot-jobs.

When running on a cluster or grid environment, the submitted jobs can be monitored, queried and, if necessary, re-submitted. The latter means that if the pipeline crashes during execution, a simple re-submission will resume the execution from the point where it stopped. Moreover, all output results are saved in temporary files and only after the analysis of each step is successfully completed are the temporary files copied to the expected results location. This ensures that even if a failure happens during the saving of the results files, the user will not end up with erroneous or incomplete files. Re-submission always generates new temporary files. After submission, the user receives information on how to access the temporary files, the job outputs and the submission scripts.

Installing all the required tools takes approximately 2 min on an Amazon EC2 virtual machine instance (t2.small) and 30 min to download and convert the GIANT.phase1_release_v3.20101123 version of the 1,000 Genomes Project. After that, the computational time needed for imputation is as published by the authors of the IMPUTE2 tool [9]. Detailed installation and setting up instructions that cover Amazon EC2 and other computing environments can be found in Additional file 1.

### Evaluation

To evaluate the computation requirements of MOLGENIS-impute, we ran the pipeline in all possible instances of Amazing Elastic Compute Cloud. We used the sample study and reference panel presented above, which is included in the tools and datasets that MOLGENIS-impute initially installs. This analysis resulted in 88,650 imputed markers. The results are shown in Table 2 and reveal that phasing requires less than 10 s for Step 1, 5 min and 23 s for step 2, and 6 min and 40 s for Step 3. Table 2 also includes a cost estimation given the current Amazon EC2 prices (November 2014). According to IMPUTE2 documentation, when the study panel is pre-phased (like in our pipeline), the imputation scales linearly with both the number of imputed markers and the number of samples. Hence, the cost presented in Table 2 can be easily extrapolated for larger datasets on various EC2 instances. Our data show that low to medium instance types exhibit optimal cost benefit.

**Table 2:** Time required to perform imputation

Instance type	ECUs	vCPUs	Memory (GiB)	Cost/hour	Phasing	Imputation
t2.small	Variable	1	1	$0.026	5′ 23″	6′ 40″
t2.medium	Variable	1	2	$0.052	4′ 28″	5′ 56″
m3.medium	3	1	3.75	$0.070	3′ 50″	5′ 50″
m3.large	6.5	2	7.5	$0.140	3′ 16″	5′ 33′
m3.xlarge	13	4	30	$0.280	1′ 0″	5′ 26″
c3.large	7	2	3.75	$0.105	3′ 4″	5′ 11″
c3.xlarge	14	4	7.5	$0.210	1′ 35″	5′ 6″
c3.2xlarge	28	8	15	$0.420	0′ 56″	5′ 5″

We evaluated MOLGENIS-impute on various Amazon EC2 instances using the presented sample study (100 samples of HapMap2 v. 3, 4,836 markers in chromosome 1) and reference panel (1,092 samples of the GIANT release of the 1,000 Genomes Project reference panel limited to 88,650 SNPs in positions from 1 to 15 million of chromosome 1). All runs used standard parameters. The results show that use of Amazon EC2 low to medium instances is quite cost-effective. According to the authors of IMPUTE2, the imputation scales linearly for number of samples/markers, so the cost can be estimated for larger datasets. ECU is EC2 Compute Unit, a relative measure of the processing power of an EC2 instance.

### Applications

MOLGENIS-impute has been used as the main imputation platform for the Genome of the Netherlands (GoNL) project [31]. GoNL is a whole-genome-sequencing project in a representative population sample, consisting of 250 trio-families from all the provinces in the Netherlands. It aimed to characterize DNA sequence variation in the Dutch population [32]. An initial study assessed the performance of GoNL as a novel reference panel for European samples [33]. Another aim of this project was to provide a population specific imputation panel for various Dutch cohorts in order to improve GWA and meta-analysis studies. Studies that have now been imputed using MOLGENIS-impute include: Dutch Prospective ALS Study [34] with 192 samples, Rotterdam Study [35] with 9,878 samples, Cohort on Diabetes and Atherosclerosis Maastricht (CODAM) [36] with 574 samples, the National Twin Registry, Amsterdam (NTR) [37] study with 1,700 samples, the LifeLines [38] study with 13,707 samples and the Leiden Longevity Study (LLS) [39] with 1,918 samples. These experiments gave us the opportunity to fine-tune our pipeline and we received valuable feedback from a diverse group of bioinformaticians.

## Conclusions

The main deliverable of our approach is a single script that downloads, configures, installs and runs all the tools, data and scripts necessary for genotype imputation. The pipeline management tool that we use ‘under the hood’ is MOLGENIS-compute, which generates scripts ready for submission to grid, cluster or local computation environments.

MOLGENIS-impute is intended for bioinformaticians and geneticists who want to minimize the time and effort needed to set up and configure an imputation pipeline that includes all the necessary quality check and data management steps. This approach belongs to the family of open bioinformatics solutions suited for HPC environments. As demonstrated, computationally intense solutions need to have gateways for environments like the cloud [40, 41] and grid [42] in order to be directly executable.

No special set up for the execution or programming language knowledge is required. The format for parameters and workflows is CSV. Simplicity and expandability was a primary development goal [43]. In this way, MOLGENIS-impute can easily act as a component of more complex genetic pipelines.

Currently, the presented pipeline supports a subset of available imputation software. Our priority was to offer a tightly coupled and tested pipeline that utilizes well-known tools. Nevertheless, additional tools like BEAGLE and MaCH/Minimac can expand the functionality and cover more uses. Adding and editing tools or computational steps in the pipeline is straightforward for a bioinformatician and is covered in the online documentation of MOLGENIS-compute [44]. Some additional effort is needed in order to adapt the presented python wrapper to these potential additions. Easing modifications in the python wrapper and extending the list of computational environments of MOLGENIS-compute is one of our future aims. More importantly, we plan to upgrade the pipeline when new imputation best practices appear.

## Availability and requirements

The source code, documentation, installation instructions and requirements are available in the following github repository:

https://github.com/molgenis/molgenis-imputation.

License: Simplified BSD License.

## Additional files

Additional file 1: MOLGENIS-impute system requirements. Additional file 2: Computation of IMPUTE2’s info metric.

## Abbreviations

CSV: comma separated values
ECU: EC2 compute unite
GWAS: genome-wide association studies
HPC: high performance computing
LD: linkage disequilibrium
NGS: next generation sequencing
PBS: portable batch system
SNP: single nucleotide polymorphism
