# Supplementary material for: Molgenis-impute: imputation pipeline in a box
Source: BMC Res Notes. 2015 Aug 19;8:359. doi: 10.1186/s13104-015-1309-3 (PMC4541731; doi:10.1186/s13104-015-1309-3)
Supplement: Additional file 1: — MOLGENIS-impute system requirements. [file 13104_2015_1309_MOESM1_ESM.docx]

**Additional file 1**

**MOLGENIS-impute system requirements.**

In order to run MOLGENIS-compute the following tools and libraries are required:

**System**: 64bit architecture. Linux Operating System (Kernel 2.6 or newer)

**Software:**

- wget (or curl)
- tar
- unzip
- bunzip2
- git
- g++
- java 1.6 or higher
- python 2.7 or higher (but not python 3.x.x)
- numpy

These tools can be installed with a simple command in a Linux Ubuntu system (require superuser privileges):

sudo apt-get install -y git openjdk-6-jre g++ python-numpy unzip make zlib1g-dev

This command also covers installation in an Amazon EC2 Ubuntu instance.

You may need to run an update before installing these tools. This can be done with the following command:

sudo apt-get update

For python installation we recommend the use of Anaconda python distribution [Anaconda]. Anaconda is a one-package python bundle that includes python 2.7, numpy and more than one hundred other scientific packages.

**References**

[Anaconda] <https://store.continuum.io/cshop/anaconda/>

Direct download link:

<http://09c8d0b2229f813c1b93-c95ac804525aac4b6dba79b00b39d1d3.r79.cf1.rackcdn.com/Anaconda-1.9.2-Linux-x86_64.sh>
