# Supplementary material for: Molgenis-impute: imputation pipeline in a box
Source: BMC Res Notes. 2015 Aug 19;8:359. doi: 10.1186/s13104-015-1309-3 (PMC4541731; doi:10.1186/s13104-015-1309-3)
Supplement: Additional file 2: — Computation of IMPUTE2’s info metric. [file 13104_2015_1309_MOESM2_ESM.docx]

**Additional file 2**

Computation of IMPUTE2’s info metric [Marchini 2010 S3]

Suppose a marker M that contains two possible alleles: A and B. Moreover suppose that this marker has been imputed in a study panel consisting of S samples. We define p_i,AA_ , p_i,AB_ and p_i,BB_ the posterior imputation probabilities for the three possible genotypes AA, AB and BB for sample i ∈ {1..S}

The Imputation Allele Frequency (IAF) of marker M is defined as:

$${IAF}_{M}=\frac{\sum_{i=1}^{S} \left( 2p_{i,BB}+p_{i,AB} \right)}{2*S}$$

The IMPUTE2 imputation info score for marker M is defined as:

$${info}_{M}=\left\{ \begin{aligned} 1-\frac{\sum_{i=1}^{S} \left( \left( p_{i,AB}+4p_{i,BB} \right)-\left( p_{i,AB}+2p_{i,BB} \right)^{2} \right)}{2*S*{IAF}_{M}*\left( 1-{IAF}_{M} \right)}, &{IAF}_{M}\in(0,1) \\ 1, &{IAF}_{M}=1, {IAF}_{M}=0 \end{aligned} \right.$$

**References**

[Marchini 2010 S3] Jonathan Marchini & Bryan Howie. Genotype imputation for

genome-wide association studies. Nature Reviews Genetics 11, 499-511 (July 2010)

<http://www.nature.com/nrg/journal/v11/n7/extref/nrg2796-s3.pdf>
